# Zinc Nanoparticles Enhance Brain Connectivity in the Canine Olfactory Network: Evidence From an fMRI Study in Unrestrained Awake Dogs

**DOI:** 10.3389/fvets.2018.00127

**Published:** 2018-07-02

**Authors:** Bhavitha Ramaihgari, Oleg M. Pustovyy, Paul Waggoner, Ronald J. Beyers, Chester Wildey, Edward Morrison, Nouha Salibi, Jeffrey S. Katz, Thomas S. Denney, Vitaly J. Vodyanoy, Gopikrishna Deshpande

**Affiliations:** ^1^Auburn University MRI Research Center, Department of Electrical and Computer Engineering, Auburn University, Auburn, AL, United States; ^2^Department of Anatomy, Physiology and Pharmacology, Auburn University, Auburn, AL, United States; ^3^Canine Detection Research Institute, Auburn University Auburn, AL, United States; ^4^MRRA Inc., University of Alabama at Birmingham, Euless, TX, United States; ^5^MR Research and Development, Siemens Healthcare, Malvern, PA, United States; ^6^Department of Psychology, Auburn University, Auburn, AL, United States; ^7^Alabama Advanced Imaging Consortium, Auburn University and University of Alabama Birmingham, Birmingham, AL, United States; ^8^Center for Health Ecology and Equity Research, Auburn University, Auburn, AL, United States

**Keywords:** zinc nanoparticles, fMRI, canine, dog, brain connectivity, olfactory system

## Abstract

Prior functional Magnetic Resonance Imaging (fMRI) studies have indicated increased neural activation when zinc nanoparticles are added to odorants in canines. Here we demonstrate that zinc nanoparticles up-regulate directional brain connectivity in parts of the canine olfactory network. This provides an explanation for previously reported enhancement in the odor detection capability of the dogs in the presence of zinc nanoparticles. In this study, we obtained fMRI data from awake and unrestrained dogs while they were being exposed to odorants with and without zinc nanoparticles, zinc nanoparticles suspended in water vapor, as well as just water vapor alone. We obtained directional connectivity between the brain regions of the olfactory network that were significantly stronger for the condition of odorant + zinc nanoparticles compared to just odorants, water vapor + zinc nanoparticles and water vapor alone. We observed significant strengthening of the paths of the canine olfactory network in the presence of zinc nanoparticles. This result indicates that zinc nanoparticles could potentially be used to increase canine detection capabilities in the environments of very low concentrations of the odorants, which would have otherwise been undetected.

## Introduction

Olfactory capability in canines is far superior to most known animals including human beings. This is in part due to the anatomical features responsible for the initial events in olfaction (1–3). The area occupied by the olfactory epithelium in human is ~3 cm^2^, while the dog (German Shepherd) has a more than 50 times larger olfactory epithelium of 170 cm^2^ (4–6). Humans have 50 million olfactory receptor neurons (ORNs), but dogs have 2 billion olfactory neurons, and dogs sniff 10 times faster than humans (7–9).

Utilization of dogs for detecting different materials in the environment is owed to this long established fact. Human society has successfully detected and evaded dangers in war zones, airports and terrorist targeted public places because dogs have been helping us with detecting explosives (10). Apart from this they have also helped us control drug/narcotics trafficking, tracking people (11). Other detection methods for explosives (12) also exist and have been proved to be effective in controlled lab environments, but sniffer dogs still have been the most effective method for this purpose outside the laboratory (10, 13–16). However, one should note that though sniffer dogs are an effective solution, they are not without stumbling blocks. One of the main hindrances is the concentration of the odorant (17) in the environment.

The process of olfaction starts with the chemical interreaction between the odorant molecules and receptor proteins in the nose. This means that detection accuracy is restricted by the concentration of the odorant present in that environment (17). In many real scenarios, target odor concentrations can even be below the dog's detection threshold. Therefore, other ways of enhancing odor-related response in the dogs are being actively investigated. Specifically, presence of nanoparticles of different metals such as copper, gold, silver, zinc, etc. are being researched. The results have mostly been unfruitful but for those with zinc. Studies have shown that the presence of zinc nanoparticles might enhance odorant responses of ORNs *in vitro* (18–20) as well as enhance functional Magnetic Resonance Imaging (fMRI)-based activation in the dog brain *in vivo* (21).

**Figure 1:**
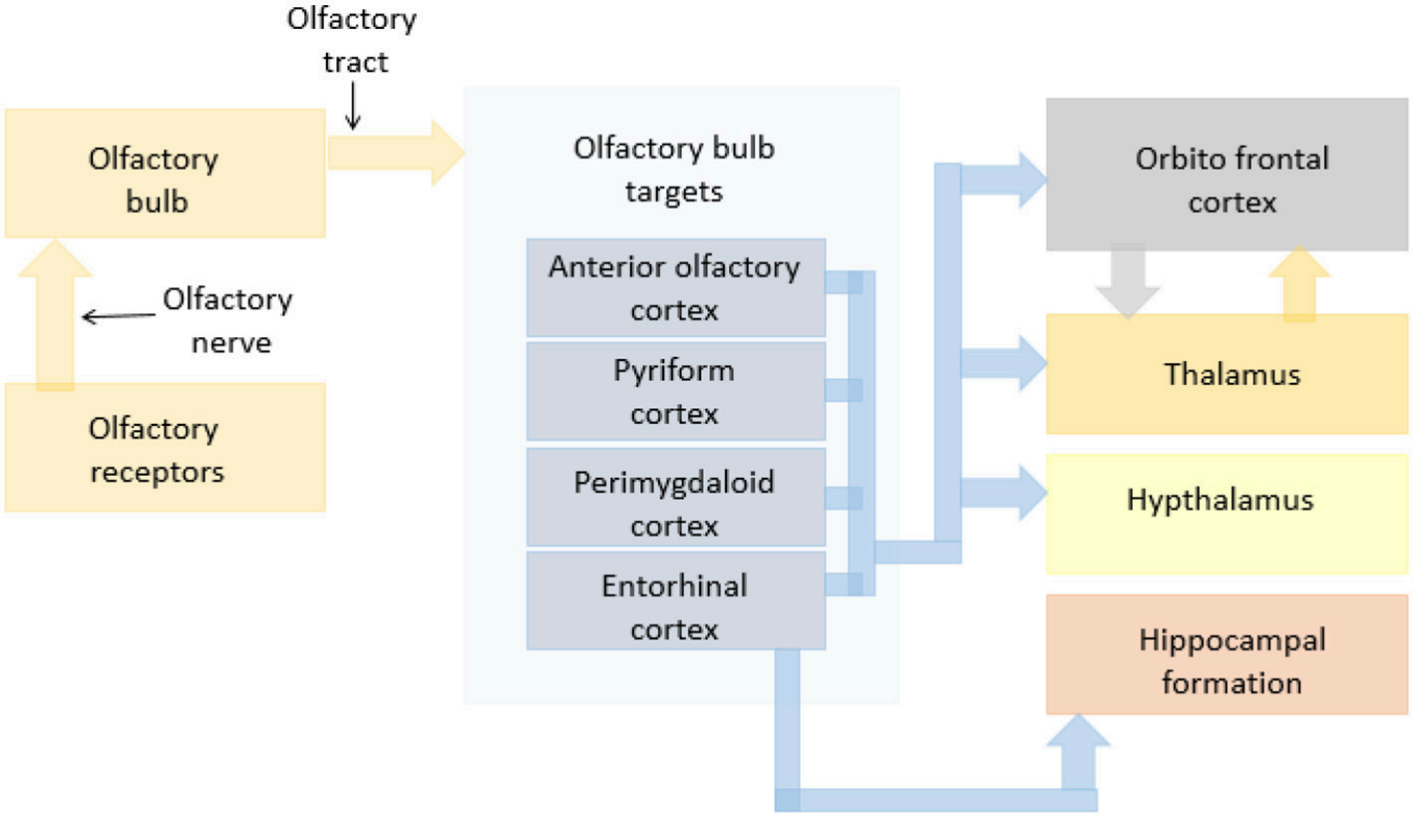
A schematic flowchart indicating olfactory pathways in dogs.

Basic olfaction as a process can be explained broadly in the sub events of sniffing, chemical binding of the odorant, signal transmission, recognition and interpretation. Each of these events involve different parts of the olfactory system (22–24). The olfaction process starts with sniffing which involves olfactory neuroepithelium of the nasal cavity. This enables the transfer of odorant molecules into the nose and to the mucus layer covering the olfactory epithelium (25). Next, the chemical binding of the odorant with a receptor protein (26, 27) initiates an intracellular cascade of signal transduction events of the G-protein-dependent adenilyl cyclase production of second messenger molecules (28) followed by opening of ion channels and passing of ion currents (29). This generates an action potential in the ORNs (30) that is projected to the olfactory bulb (OB) (31). The signal thus generated is transmitted to the regions of pyriform cortex, periamygdaloid cortex, and entorhinal cortex through olfactory stria. From pyriform cortex and periamygdaloid cortex, the signal is then transmitted to the thalamus and frontal cortex, where it is recognized and interpreted (32, 33). The regions of the Hippocampus receive the signal from entorhinal cortex for recognition purposes as well (34, 35). Apart from these, various regions of the brain such as the amygdala are involved in the emotional processing resulted from the odors recognized. A schematic of the olfactory pathway in dogs reconstructed based on previous literature is shown in Figure 1.

Based on previous *in vitro* (18, 20, 36) and *in vivo* (21) studies, we concluded that olfactory enhancement by the zinc nanoparticles is composed of two components. One component is based at the level of olfactory sensory receptors, and the second part of the olfactory enhancement is positioned at higher levels of olfactory perception. The first part was explained by a simple model: The endogenous zinc nanoparticles produce a certain number of functional receptor dimers that can be triggered by the odorant as well as take part in the generation of the olfactory signal. When the olfactory epithelium is subjected to a mixture of zinc nanoparticles and also the same odorant, extra receptor dimers are created by joining with each other pairs of previously unbound receptors (21). In this study, we investigate the second part of olfactory enhancement by zinc nanoparticles. We test the hypothesis that the connectivity of between brain regions that are situated above the olfactory sensory neurons have increased strengths in the presence of zinc nanoparticles.

We obtained the strength of paths between olfaction-related brain areas for the condition of dogs being exposed to odorant with zinc nanoparticles and compared them to those obtained for odorants without nanoparticles. We used two additional control conditions: a suspension of zinc nanoparticles in water vapor and just water vapor.

## Materials and methods

### Preparation of dogs

A total of 8 dogs, raised in the Auburn University Canine Performance Sciences Program, with ages between 12 and 60 months were used for this experiment. Ethical approval for the study was obtained from the Auburn University Institutional Animal Care and Use Committee. We confirm that all methods were performed in accordance with the relevant guidelines and regulations. The concentrations of the zinc nanoparticles the dogs were exposed to are non-toxic to them (37) thus their use is not unethical in this study. The amount of zinc exposure from the sniffing is calculated as follows: the test concentration of metallic zinc in the test solutions was 0.02 nM, or 1.3 ng/L. The approximate volume of the solution applied per pulse (sniff) is 0.010 mL. For 5 sniffs per run, the volume of the solution is 0.05 mL. The daily dog exposure does not exceed 10 runs. Therefore the testing volume of zinc nanoparticle suspension does not exceed 0.5 mL. Thus, the amount of estimated zinc inhaled by a 30 kg dog per day is less than 0.5 × 10^−3^ L × 1.3 ng/L = 6.5 × 10^−7^ microgram/dog/day. The daily recommended amount of zinc per day for the 30 kg average body weight dog is 30 mg, or 3 × 10^4^ microgram (37). This level of zinc intake is 50 billion times higher (3 × 10^4^/6.5 × 10^−7^) than daily exposure during fMRI experiments. Additionally, we have previously demonstrated that zinc nanoparticles are cleared from olfactory epithelium within 10 s (20). Also, zinc nanoparticle at the level we used in our work do not destroy olfactory epithelium in contrast to the zinc sulfide that is known to damage olfactory epithelium (38).

These dogs were trained to remain in the scanner bed with their heads inserted into the human knee coil (in prone position) for the duration of the scanning, carried out while the dogs were awake and unrestrained. Positive reinforcement behavior shaping procedures were used to keep them as still as possible and to desensitize them to the loud scanner noise.

### Odorants

The odorant used in the experiment was a mixture of ethyl butyrate, eugenol, and (+) and (−) carvone in water at a concentration of 0.016 mM. This is well above the dog's LOD (level of detection) in air for odorants we used, which has been shown to be at the level of 5 pM (10^−12^ M) (39). This odorant mixture, as well as the training procedure, were the same as in Jia et al. (40). The odorant concentration was considered to be 0.016 mM as it was the low concentration in the previous work (40), for which the activation of olfaction related areas in the dog's brain could be detected. Nevertheless, we were able to detect a significant increase in activation when a higher concentration (0.16 mM) was utilized in that study. Saturation of the EOG signal takes place only at ~10 mM of the same odorant mixture (20). These data reveal that using a low odorant concentration of 0.016 mM in the current work, there is sufficient dynamic range for zinc nanoparticles to enhance olfaction related activation in the brain without saturating the brain responses. It has been shown that the spatial clustering of principal responses to the individual odorants of this mixture show statistically distinct and different glomerular patterns (41). This fact may potentially enhance the odorant presentation in fMRI tests.

The concentration of odorant is given in the water solution. Because the water/air partition coefficient for all odorants we used in our experiments is very low (~10^−4^), the concentration of the odorants in the head space is in parts per billion range. For example, the concentration of Eugenol in head space can be estimated using Amoore-Buttery equation for the water/air partition coefficient, K_aw_, from value of vapor pressure, solubility in water and molecular weight (42):

$$\begin{array}{l} K_{aw}=\left(\left(\frac{55.5}{S-0.0555}\right)\times M+1\right)\times P\times 0.97\times 10^{-6} \end{array}$$

where *P* is vapor pressure in mm Hg, *S* is solubility in water in g/L of the pure odorant at 25°C and *M* is its molecular weight. For Eugenol, we have *P* = 0.0226 mm Hg; *S* = 2.47 g/L; *M* = 164.2 g/mol. According to the Amoore-Buttery equation, *K*_*aw*_ = 8.08 × 10^−5^. This value of *K*_*aw*_ for Eugenol agrees well with that obtain experimentally (43).

Thus, the concentration of Eugenol in head space (and consequently delivered to a dog) equals to C_h_ = K_aw_ × C_b_ = 8.08 × 10^−5^ × 0.016 × 10^−3^ M = 1.3 × 10^−9^ M, where C_h_ is a head space concentration and C_b_ is balk concentration in liquid. The head space concentration can be converted to nM and ppb as follows.

C_h_ = 1.3 × 10^−9^ M = 1.3 nM

C_h_ = (Mass in m^3^)/molecular mass) × (volume of 1 mole) = (9.5 μg/m^3^/164.2 g/mol) × 24.45 = 1.4 ppb.

### Zinc nanoparticles

The procedure of obtaining and mixing of the zinc nanoparticles was similar to that described in Jia et al. (21). The zinc nanoparticles were prepared by the underwater electrical discharge method as shown in Vodyanoy et al. (44). The produced particles were centrifuged at 20,000 × g for 1 h at 8°C. After centrifugation, the pellet is discarded and the supernatant is subjected to further centrifugations at 47,000 g for 1 h at 5°C to produce a fraction of nanoparticles enriched in particles of 1–2 nm. The particle physical properties were analyzed by electron microscopy, atomic force microscopy, and X-ray photoelectron spectroscopy (44). The total concentration of metal in suspension was measured by atomic absorption spectra (GTW Analytical Services, Memphis, TN, USA). Zinc nanoparticles had crystalline structure with an average diameter of 1.2 ± 0.3 nm. About 94% of metal atoms were not oxidized. The zinc nanoparticles were suspended in odorant solution at concentration of 0.02 nM.

### Data acquisition

The data acquisition procedure was described in detail in our previous publications (21, 40). Briefly, it consisted of: a 3T MAGNETOM Verio scanner (Siemens Healthcare, Erlangen, Germany), a 15 channel human knee coil adapted as a dog head coil, customized odorant applicator for computer-controlled delivery and evacuation of odorant stimulus, mask for receiving the odorant stimulus and covering the nose and mouth of the dogs, an external infra-red camera used to track head motion in dogs and retrospectively correct for motion artifacts in the data. Functional MRI data was obtained using an EPI (Echo-planar Imaging) sequence with the following parameters: repetition time (TR) = 1,000 ms, echo time (TE) = 29 ms, field of view (FOV) = 192 × 192 mm^2^, flip angle (FA) = 90 degree, in-plane resolution 3 × 3 mm^2^, in-plane matrix 64 × 64, and whole brain coverage. Anatomical data was obtained for registration purposes using an MPRAGE sequence with the following parameters: TR = 1,550 ms, TE = 2.64 ms, voxel size: 0.792 × 0.792 × 1 mm^3^, FA = 9°, in-plane matrix = 192 × 192, FOV = 152 × 152 mm^2^, number of slices: 104.

Data was obtained for each dog while being exposed to the following set of odorants: Odorants+ zinc nanoparticles (OZ), odorants alone (O), water vapor + zinc nanoparticles (WZ), water vapor alone (W). Each scanning session included 1 run of structural scan, 2 runs of functional scans involving odor stimulation with zinc nanoparticles, 2 runs with odorant alone, 2 runs of functional scans involving exposure to zinc nanoparticles alone in water vapor, and 2 runs of functional scans involving exposure to water vapor alone. These functional scans were run in random order for each dog.

### Experimental paradigm

**Figure 2:**
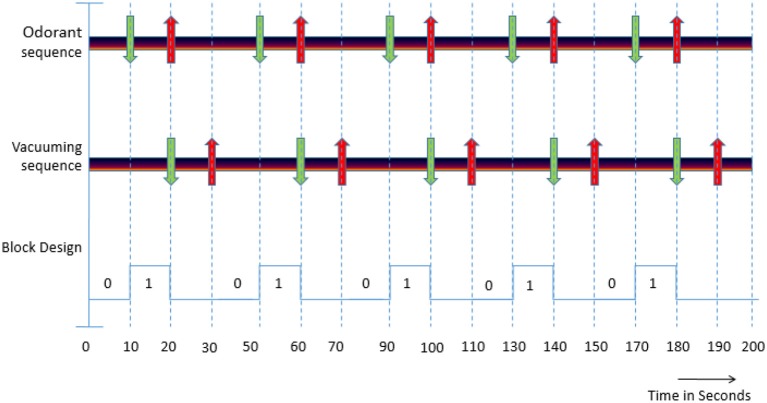
(i) Odorant sequence: Green down arrow indicates the starting of the stimulant presentation and red up arrow indicates ending of the stimulation. (ii) Vacuuming sequence: Green down arrow indicates the starting of the vacuuming to remove odorant and the red up arrow indicates ending of vaccuuming. (iii) Block Design: “0” indicates the absence of an odorant (OFF condition) and “1” indicates the presence of an odorant (ON condition).

As described in Jia et al. (21), each functional run with odorant stimulus had 5 blocks of odorant exposure each lasting for 10 s followed by 30 s of rest block to prevent the adaptation of the dog's olfactory response to the odorant (Figure 2). The stimulus block involved pumping of the odorant to the mask so as to expose the subject to it. The resting blocks consisted of an initial 10 s for vacuuming the odorant from the pipes and the mask followed by 20 s of no stimulation. Each run lasted for 200 s with the onset times of the stimulant in each run for the 5 blocks being 10, 50, 90, 130, and 170 s, respectively. The choice of 10-s odor-on condition and 30-s odor-off paradigm was guided by previous studies showing that it is effective for eliciting measurable neural response while preventing habituation (21).

A schematic of the experimental paradigm is shown in Figure 2 and can be explained as follows. In the odorant sequence, green arrows indicate the onset time of the odorant stimulus in the 4 conditions (pure odorants, odorants + zinc nanoparticles, pure water vapor, and water vapor + zinc nanoparticles) and down arrows indicate the time when the stimulation ends. The four conditions above were presented randomly across runs within a session. In the vacuuming sequence, the green arrows indicate the beginning of the vacuuming or clearance of odorant, and red arrows indicate the ending. The block design represents the paradigm with “0” indicating absence of stimulus (OFF condition) and “1” denoting the presence of odorant (ON condition).

### Data processing

As described in Jia et al. (40), preprocessing of fMRI data was done using the software SPM8 (http://www.fil.ion.ucl.ac.uk/spm/software/spm8/, Functional Imaging Lab, The Welcome Trust Centre for NeuroImaging, in the Institute of Neurology at University College London). The basic steps of slice timing correction, realignment to the first functional image, spatial normalization to a template defined by us as in Jia et al. (21, 40), and spatial smoothing were done. Then the preprocessed fMRI data was input to a general linear model (GLM) and statistical tests were performed for obtaining voxels in the canine brain which were activated for the comparison of odorants + zinc nanoparticles with each of the following conditions: zinc nanoparticles alone, water vapor + zinc nanoparticles, water vapor alone, were obtained. Voxels significantly active in all of the following conditions, i.e., (odorants + zinc nanoparticles > zinc nanoparticles alone) n (odorants + zinc nanoparticles > water vapor + zinc nanoparticles) n (odorants + zinc nanoparticles > water vapor alone), were identified and used for definition of ROIs as discussed below. The GLM also modeled variance from confounding factors such as time and dispersion derivatives (in order to model the variability of the hemodynamic response function), motion parameters obtained from realignment, as well as motion parameters obtained from the external camera-based motion tracking device. We showed that adding zinc nanoparticles to a single low concentration of odorant, increases amplitude of the output signal, which is equivalent to the signal of 10 times stronger odorant (20, 21). The brain olfactory areas present a very complex connectivity system. Therefore, to analyze connectivity, we tried to keep the stimuli as simple as possible.

Considering the activations obtained from the contrast mentioned above (only the activated voxels) the following Regions of Interest (ROIs) were selected: Amygdala, Hippocampus, Olfactory bulb, Thalamus, Caudate, Pyriform lobe, Frontal cortex. While the voxels themselves were dictated by the contrast defined above, the nomenclature of the ROIs they belong to were identified using a dog atlas (45). For each of these ROIs, mean time series from activated regions were extracted for every run. These time series were then subjected to blind hemodynamic de-convolution using a cubature Kalman filter and smoother (46) to obtain the underlying latent neural variables. This was done in order to remove the confounding effect of HRF variability on connectivity results (47–53). Directional brain connectivity between the ROIs was then obtained for each condition using Dynamic Granger Causality (DGC) by using the analysis framework reported before (54–62). Connectivity for all possible paths between ROIs for the condition odorant +zinc nanoparticles were computed. Mean connectivity was also computed for each path for the conditions of odorant, water vapor + zinc nanoparticles and water vapor alone. Using two sample *t*-tests, paths whose connectivity strength was stronger for the condition of Odorant + zinc nanoparticles (OZ) as compared to other control conditions of odorant (O), water vapor + zinc nanoparticles (WZ), and only water vapor (W) were indicated.

## Results

**Figure 3:**
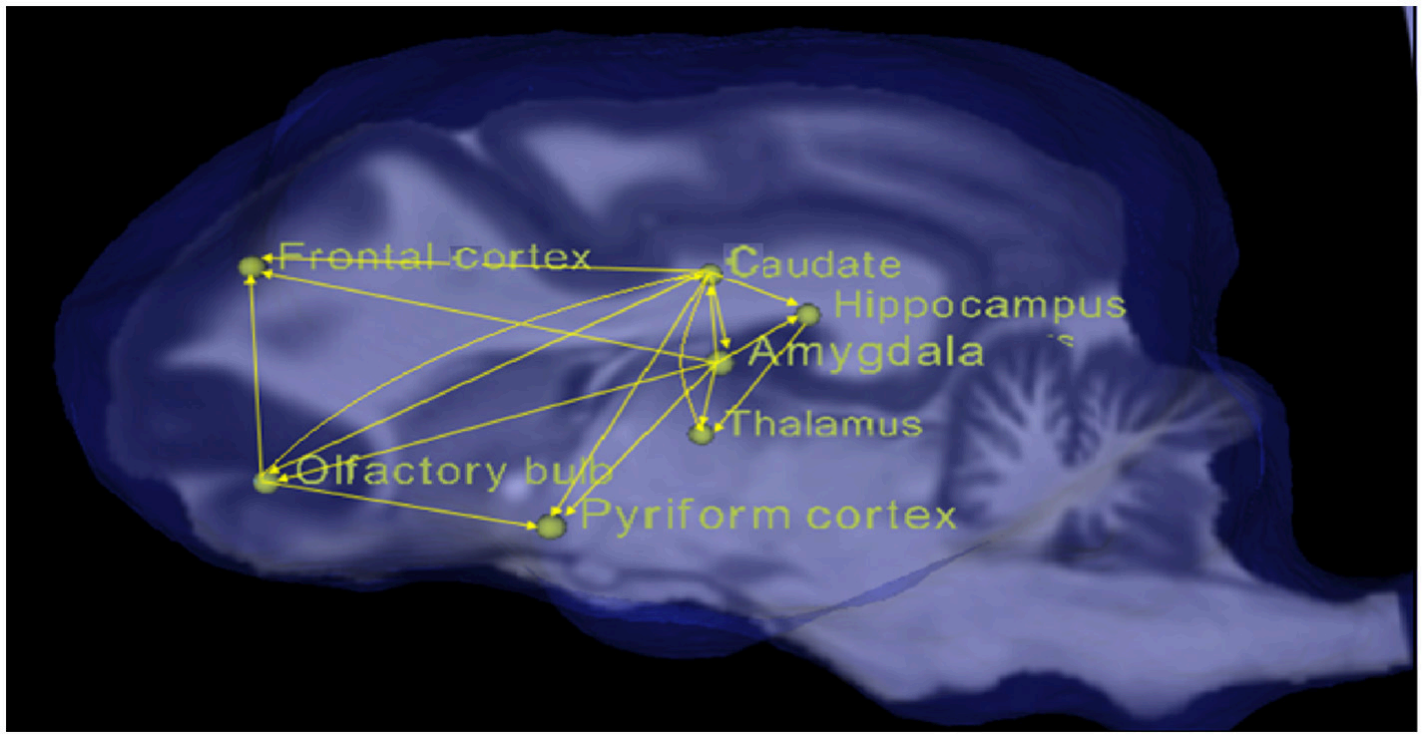
Pictorial depiction of paths with significant increase in connectivity strength for the condition of odorant + zinc nanoparticles (OZ) compared to conditions of odorant (O), water vapor+ zinc nanoparticles (WZ), and water vapor alone (W).

All the paths with corrected *p* < 0.05 for the condition of Odorant + Zinc nanoparticles greater than the conditions of Odorant, water vapor + zinc nanoparticles, water vapor (OZ > O, WZ, W) were obtained and are listed in the Table 1 along with their connection strengths. The paths are also shown pictorially depicted in Figure 3. It can be seen that many paths within the dog olfactory network show strengthening in the presence of zinc nanoparticles. When similar results were generated using different random splits of the data, the significant paths did not change. This provides some reassurance that the results are replicable.

**Table 1:** Paths with significant increase in connectivity strength for the condition of odorant + zinc nanoparticles (OZ) compared to conditions of odorant (O), water vapor+ zinc nanoparticles (WZ) and water vapor alone (W).

**Path origin**	**Path termination**	***P*-value**	**Mean connectivity**	
			**OZ**	**O, W, WZ**
Amgdala	Caudate	8.95 × 10^−24^	0.252	0.052
Amgdala	Hippocampus	3.23 × 10^−11^	0.194	0.067
Amgdala	Olfactory bulb	1.80 × 10^−07^	0.159	0.056
Amgdala	Pyriformlobe	3.18 × 10^−21^	0.254	0.06
Amgdala	Thalamus	5.79 × 10^−18^	0.23	0.066
Amgdala	Frontal cortex	2.26 × 10^−12^	0.217	0.072
Caudate	Amgdala	6.16 × 10^−20^	0.204	0.053
Caudate	Hippocampus	1.74 × 10^−23^	0.194	0.013
Caudate	Olfactory bulb	2.13 × 10^−25^	0.194	0.038
Caudate	Pyriformlobe	5.13 × 10^−18^	0.213	0.038
Caudate	Thalamus	7.45 × 10^−11^	0.187	0.042
Caudate	Frontal cortex	2.13 × 10^−21^	0.156	0.061
Hippocampus	Thalamus	4.56 × 10^−2^	0.218	0.106
Olfactory bulb	Caudate	0.27 × 10^−2^	0.136	0.114
Olfactory bulb	Pyriformlobe	1.53 × 10^−2^	0.162	0.119
Olfactory bulb	Frontal cortex	0.04 × 10^−2^	0.154	0.099

*Resultant p-value of the t-test, mean connectivity values of the paths for conditions OZ and (WZ, W, O) are shown*.

**Figure 4:**
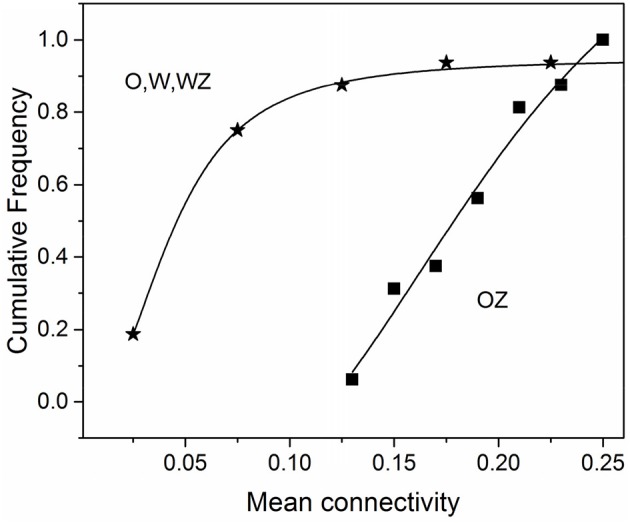
Cumulative frequency distribution of the mean connectivity for the condition of odorant + zinc nanoparticles (OZ) compared to conditions of odorant (O), water vapor+ zinc nanoparticles (WZ), and water vapor alone (W). Data were taken from Table 1.

Our previous fMRI analysis of the olfactory system in conscious dogs showed that an increase of odorant concentration of 10 times caused a considerable escalation of brain activity manifested by the growth of the total number of activated voxels from 379 to 759, at the ratio of 2.0 (40). When zinc nanoparticles were added to the odorant, we observed the doubling of the total number of activated voxels (21), which is equivalent to the activation obtained by a 10-folds higher concentration of odorant. In this work, we documented the robust increase in connectivity strength for odorant with zinc nanoparticles compared to the odorant alone, water vapor with zinc nanoparticles, and water vapor alone (Table 1). The mean value of connectivity increase was 3.14 ± 1.53 (SD) (*n* = 16), which was consistent with the ~3-fold increase of electro-olfactogram (EOG) amplitude evoked by a 10-fold increase in odorant concentration in rodents (20, 36), and the brain activity increase observed in dogs (21, 40). Analysis of the cumulative frequency distributions (Figure 4) shows a ~3-fold shift to larger values of connectivity in the presence of zinc nanoparticles.

## Discussion

Canine olfaction has been very useful to mankind over decades for various tasks such as detecting explosives, people etc. However, they still do not seem to be accurate on occasions due to reasons such as the low concentrations of the odorant in the surrounding environment. Therefore, understanding the olfactory system in canines and methods of enhancing their olfactory capabilities are of high interest. Efforts have been made in this direction using *in vitro* cellular (63, 64) or behavioral approaches (65–68. *In vivo* imaging studies till now have mostly concentrated on activations in various regions of the brain (21, 40). Given the strides made in human imaging for gaining a perspective on brain function using connectivity modeling in the brain, our study is an attempt (likely the first, in awake dog imaging) to explore the canine olfactory system and its enhancement with zinc nanoparticles using connectivity modeling.

Perceived odor intensities by humans are observed to be highly correlated with the EOG amplitude (69). EOG studies indicate that neural activity at the human olfactory epithelium mirrors olfactory perception (70). Since its introduction, fMRI has become a very powerful instrument to noninvasively infer underlying mechanisms of brain function (71). Our prior work has demonstrated the use of fMRI for inferring the cognitive foundations of odor processing in fully conscious and unrestrained dogs (40).

Our hypothesis, that the connectivity of the various signal paths involved in the process of olfaction will increase in the presence of the zinc nanoparticles, is motivated by previous works which have shown *in vitro* enhancement of the olfactory response in olfactory sensory neurons in the presence of zinc nanoparticles (18, 20, 36, 72) as well as *in vivo* enhancement observed in terms of increased fMRI-based activation of olfaction-relevant regions of the dog brain (21). The sensory olfactory nervous system is a part of the peripheral somatic nervous system and transmits olfactory signals from olfactory sensory neurons to the brain. Using whole cell patch clamp, we demonstrated that zinc nanoparticles significantly increase electrical signals from individual neurons (20). Below, we discuss our results in the context of what we already know about the canine olfactory system.

Electrical potentials measured in the ORNs (73, 74) at the initiation of the olfaction are proportional to the logarithm of the concentrations of odorants (75, 76). The olfactory bulb, as described before, receives the signal from the receptor neurons (31) and transmits them to the amygdala, entorhinal cortex and pyriform cortex. The signal received by the olfactory bulb and further transmitted to the above regions is directly related to the odorant molecules reacting with the receptor neurons. We can observe from the results that the paths originating from the olfactory bulb and driving to the pyriform lobe and entorhinal cortex significantly increased their strength in the presence of zinc nanoparticles. The amygdala mainly contributes to the processing of the emotionally salient content in the olfactory stimuli (77). We observed that all the paths originating from and towards the amygdala had enhanced connectivity in the presence of zinc nanoparticles. The caudate, in conjunction with the amygdala and hippocampus, participates in functions related to memory, goal oriented activities, and emotions i.e., they are involved in the higher order processing of the olfactory stimuli (34, 35). In addition, the frontal cortex is known to be involved in the interpretation and recognition of olfactory stimuli (32, 33) while the thalamus acts as a relay between cortical and sub-cortical structures in the olfactory network. Our results show a tight network of paths between the frontal cortex, thalamus, caudate, amygdala, and hippocampus whose connectivity was enhanced in the presence of zinc nanoparticles.

It is interesting to note that in our previous report (40), we had demonstrated that when the odorant concentration increased 10 times it caused the spatial extent of activation in conscious dogs to approximately double in area (40). This was corroborated in a followup study using zinc nanoparticles wherein the addition of nanoparticles to the odorant increases the spatial extent of activated region 2-fold (21). The finding indicates that zinc nanoparticles may be equivalent to a 10-fold increase in odor concentration. Analysis of connectivity data in the presence of zinc nanoparticles from Table 1 shows a 3-fold shift to larger values of connectivity in paths belonging to the canine olfactory network (Figure 4). This is in agreement with a similar 3-fold increase in EOG amplitude evoked by a 10-fold increase in odorant concentration in rodents (20, 36). Our data are in agreement with results obtained by direct optical recording of the activity of rat glomeruli in rat olfactory bulb (78). They described the relative activity of glomeruli as a sigmoidal function of odorant concentration. However, the major increase of activity is proportional to a logarithm of stimuli, and a 10-fold increase in odorant concentration correspond to ~3 times increase in the relative activity of glomeruli in the olfactory bulb (23). Our results agree well with those showing connectivity from olfactory sensory neurons expressing OR37 receptors into the higher brain centers visualized by genetic tracing (79).

The results of study suggest that zinc nanoparticles enhance the canine olfactory sensitivity by potentially upregulating both activity (21) and connectivity (current study) in the canine olfactory network. If corroborated by behavioral studies, this finding could provide a potential method of improving the detection capabilities of sniffer dogs in ultra-low concentration environments. The longer term implications of this work could provide an enhancement in the individual sense of smell in disorders such as Alzheimer's and Parkinson's, which show olfaction loss (80). In early Alzheimer's, olfactory deficits are a preclinical symptom that aggravates with disease progression (81, 82). Alzheimer's disease impacts ~5.5 million Americans as of 2017 and is the 10th leading cause of death in the United States (80). We hope that upcoming therapies with zinc nanoparticles functioning on the olfactory receptor level at minimal concentrations could compensate the loss of smell and improve emotional well-being as well as quality of life.

## Author contributions

GD, VV, TD, EM, and PW conceived the idea and designed the experiment. GD, NS, and RB optimized sequences and acquired the data. PW handled the dogs. OP prepared the odorants and operated the odor applicator device. CW trained GD and RB for using the external motion tracking device in addition to providing the device. GD, BR, and VV analyzed the data. GD, BR, VV, and JK interpreted the results. All authors contributed toward writing the manuscript.

### Conflict of interest statement

Two of the authors are employed by commercial companies. CW is the founder and CEO of MRRA Inc., who supplied the optical motion tracking system and contributed to this work by training the first author on using this product. MRRA Inc., is the license holder for the patent covering the motion tracking product. NS is an employee of Siemens Healthcare, Malvern, PA who is stationed at the MRI Research Center in Auburn University. She contributed by optimizing the sequences used to acquire fMRI data in this study. However, note that these authors had no role in data analysis and its interpretation. These competing interests do not alter the authors' adherence to common policies on sharing data and materials. The remaining authors declare that the research was conducted in the absence of any commercial or financial relationships that could be construed as a potential conflict of interest.
